# Seroprevalence of *Toxoplasma gondii *infection in pet dogs in Lanzhou, Northwest China

**DOI:** 10.1186/1756-3305-4-64

**Published:** 2011-05-04

**Authors:** Song-Ming Wu, Si-Yang Huang, Bao-Quan Fu, Guang-Yuan Liu, Jia-Xu Chen, Mu-Xin Chen, Zi-Guo Yuan, Dong-Hui Zhou, Ya-Biao Weng, Xing-Quan Zhu, De-He Ye

**Affiliations:** 1 State Key Laboratory of Veterinary Etiological Biology, Key Laboratory of Veterinary Parasitology of Gansu Province, Lanzhou Veterinary Research Institute, CAAS, Lanzhou, Gansu Province 730046, PR China; 2 College of Veterinary Medicine, Gansu Agricultural University, Lanzhou, Gansu Province 730070, PR China; 3 College of Veterinary Medicine, South China Agricultural University, Guangzhou, Guangdong Province 510642, PR China; 4 National Institute of Parasitic Diseases, Chinese Center for Disease Control and Prevention, Laboratory of Parasite and Vector Biology, Ministry of Health, WHO Collaborating Center for Malaria, Schistosomiasis and Filariasis, Shanghai 200025, PR China; 5 College of Animal Science and Technology, Yunnan Agricultural University, Kunming, Yunnan Province 650201, PR China; 6 College of Veterinary Medicine, Hunan Agricultural University, Changsha, Hunan Province 410128, PR China

## Abstract

**Background:**

In recent years, surveys of *Toxoplasma gondii *infection in dogs have been reported worldwide, including China. However, little is known about the prevalence of *T. gondii *in pet dogs in Northwest China. In the present study, the prevalence of *T. gondii *in pet dogs in Lanzhou, China was investigated using the modified agglutination test (MAT).

**Results:**

In this survey, antibodies to *T. gondii *were found in 28 of 259 (10.81%) pet dogs, with MAT titers of 1:20 in 14 dogs, 1:40 in nine, 1:80 in four, and 1:160 or higher in one dog. The prevalence ranged from 6.67% to 16.67% among dogs of different ages, with low rates in young pet dogs, and high rates in older pet dogs. The seroprevalence in dogs >3 years old was higher than that in dogs ≤1 years old, but the difference was not statistically significant (*P >*0.05). The seroprevalence in male dogs was 12.50% (17 of 136), and in female dogs it was 8.94% (11 of 123), but the difference was not statistically significant (*P >*0.05).

**Conclusions:**

A high prevalence of *T. gondii *infection was found in pet dogs in Lanzhou, Northwest China, which has implications for public health in this region. In order to reduce the risk of exposure to *T. gondii*, further measures and essential control strategies should be carried out rationally in this region.

## Background

*Toxoplasma gondii *is an important zoonotic intracellular protozoan parasite, which can affect all warm-blooded mammals and birds throughout the world, including humans [1,2]. *T. gondii *is transmitted by ingestion of tissue cysts from undercooked or raw meat, consumption of food or drink contaminated with oocysts, or ingestion of oocysts from the environment by accident [1]. Nearly one third of the global human population has been infected with *T. gondii*, however, infection in healthy individuals is usually asymptomatic and only a small percentage of exposed people have obvious clinical symptoms [1,3,4]. Nonetheless, if *T. gondii *infection occurs in pregnant women it can cause severe disease such as toxoplasmic encephalitis, blindness, abortion, fetal abnormalities or even prenatal death [5]. Infection of immunocompromised patients (e.g. HIV/AIDS patients) with *T. gondii *can cause acute morbidity and even death [6,7].

Pet dogs are often regarded as the faithful friends and intimate companions of humans. Unfortunately, *T. gondii *oocysts in pet dogs can traverse the intestinal tract and finally be excreted in the feces [8], which can pose a threat to human health, particularly in pregnant women and immunologically deficient people as described above. Investigations of the prevalence of antibodies to *T. gondii *in dogs have been conducted extensively in the world [9-14], however, only limited surveys of *T. gondii *infection in pet dogs have been reported. In recent years, there also have been various surveys of *T. gondii *infection in dogs in the People's Republic of China (PRC) [Table 1, [15-26]], but in Northwest China only one such investigation of pet dogs has been reported in Inner Mongolia [16].

**Table 1:** Prevalence of *T. gondii *infection in dogs in People's Republic of China

Provinces/Cities	No. tested	Positive (%)	Serologic test^d^	Cut-off value	Time tested (year)	References
Guangzhou	114^a^	17.5	ELISA	UN^e^	2007-2008	[15] Zhang et al., 2010
	36^b^	33.3				
Inner Mongolia	68^a^	7.4	ELISA	UN	2009-2010	[16] Lu et al., 2010
	35^c^	2.9				
	64^b^	23.4				
Shenzhen	598^a^	3.34	ELISA	UN	2009-2010	[17] Xie et al., 2010
Zhengzhou	106^a^	12.26	IHA	1:64	2009	[18] Zhang et al., 2010
Xinjiang	96^b^	4.17	IHA	1:64	UN	[19] Zhang et al., 2009
Shanghai	360^a^	11.67	PAPS	UN	2001-2002	[20] Gong et al., 2009
	620^a^	4.03	IHA	1:64	2003-2007	
	355^a^	4.51	PAPS	UN	2008-2009	
	355^a^	3.94	IHA	1:64	2008-2009	
Shanghai	3982^a^	13.00	PAPS	UN	2002	[21] Wang et al., 2006
Taizhou	5248^a^	0.26	UN	UN	2007-2008	[22] Lu et al., 2009
Haikou	462^a^	2.6	ELISA	UN	2007-2008	[23] Huang et al., 2008
Dongguan	610^c^	0.66	IHA	1:64	2005-2006	[24] Zhang et al., 2007
Beijing	159^a^	13.21	ELISA	UN	UN	[25] Yu et al., 2006
Hebei	78^c^	26.92	ELISA	UN	2000-2001	[26] Yuan et al., 2004

^a ^pet dogs, ^b ^stray dogs, ^c ^type not specified by others.

^d ^ELISA: enzyme-linked immunosorbent assay, PAPS: polyaledehyde polystyrene, IHA: indirect hemagglutination test

^e ^UN: unknown

The objectives of the present survey were to determine the seroprevalence of *T. gondii *infection in pet dogs in Lanzhou, the capital of Gansu province, Northwest China, and to evaluate the main associated risk factors relating to exposure to *T. gondii *in this region. In the present paper, we also summarise serological surveys of the prevalence of *T. gondii *infection in dogs in China (Table 1), because most of these surveys were originally published in local Chinese journals [16-26], which are not accessible to international readers.

## Materials and methods

### The study site

The study was conducted in Lanzhou Municipality, the capital of Gansu province, covering an area of 13,085 square meters in Northwest China. Lanzhou is situated in the geometric center of China's territory between east longitudes of 102°30" to 104°30" and north latitudes of 35°5" to 38°, which has an average height of 1,500 meters above sea level. The city is located between mountains to the north and south, and is crossed by the Yellow River from west to east, with a characteristic of ribbon basin geography. It has a typical temperate and monsoonal continental climate, with an annual rainfall of 360 mm mainly from June to September, an average annual temperature of 9.3°C, average yearly sunlight exposure of 2,446 hours and a frost-free season of about 180 days.

### Sampling of pet dogs

A total of 259 blood samples were collected from the leg veins of pet dogs between November and December 2010 in Lanzhou. These pet dogs were admitted into pet hospitals located in four districts of Lanzhou City, including Chengguan District, Anning District, Xigu District and Qilihe District, with a variety of diseases. Pet dog owners were asked for details of the animals breed, age, sex, source and medical history using a structured questionnaire. Blood samples were kept at 37°C for 2 h and centrifuged at 2,000 *g *for 5 min. The resulting sera samples were stored at -20°C until further analysis.

### Serological examination

Sera from pet dogs were diluted in a two-fold serial dilution from 1:20 to 1:320 and investigated for *T. gondii *antibodies using the modified agglutination test (MAT) as described previously [27]. MAT is a sensitive and specific technique for measuring *T. gondii *antibodies, which has been used extensively in experimentally and naturally infected dogs [11,13,28], and other animals [29]. Briefly, MAT was performed with a suspension of *Toxoplasma *tachyzoites fixed with formalin, serum samples which had been diluted in phosphate buffered saline (PBS, pH 7.2), positive and negative control sera, antigen diluting buffer containing bovine serum albumin (BSA), 2-mercaptoethanol [to deplete the sera of non-specific immunoglobulin (Ig) M antibody], and Evans blue dye solution. MAT titers of 1:20 or higher were considered as positive [12], and those sera with dubious results were re-tested. Positive and negative controls were incorporated in each test and tested at the same dilutions of sera samples. A negative result was returned when the base of the "U" bottom 96 well microtiter plates contained a blue pellet; conversely a clear bottom indicated a positive result.

### Statistical analyses

Differences in the seroprevalence of *T. gondii *infected pet dogs between male and female dogs, and different age groups were analyzed using a Chi square test in Predictive for Analytics Software (PASW^®^) Statistics 18. The *P *value < 0.05 was considered statistically significant. The correlation between the rates of infection in different age groups was calculated with Excel 2003 (Microsoft^®^).

## Results

In the present study, a total of 259 pet dogs (123 females and 136 males) from Lanzhou were examined by MAT. Out of these samples, 28 (10.81%) were seropositive for *T. gondii*. Among these positive pet dogs, seroprevalence varied in different age groups, ranging from 6.67% to 16.67% (Table 2). The investigation also showed that the prevalence in female animals was 8.94%, and 12.50% in male animals (Table 2). Table 3 indicates that antibodies to *T. gondii *were found in 28 (10.81%) of 259 pet dogs at the cut-off of 1:20, with titers of 1:20 in 14 dogs, 1:40 in nine, 1:80 in four, and 1:160 or higher in one pet dog.

**Table 2:** Prevalence of antibodies to *Toxoplasma gondii *in pet dogs by gender and age in Lanzhou, northwest China using modified agglutination test (MAT)

Biometric data	Gender		Total	Age (years)			
			
	Male	Female		≤1	2	3	>3
Sample No.	136	123	259	121	66	30	42
Positive No.	17	11	28	9	10	2	7
Prevalence (%)	12.50	8.94	10.81	7.44	15.15	6.67	16.67

**Table 3:** Antibody titers to *Toxopalsma gondii *infection in pet dogs in Lanzhou, northwest China by modified agglutination test (MAT)

Age group (years)	No. of sera with MAT titers of				
	
	1:20	1:40	1:80	1:160	≧1:20
≤1	6	2	0	1	9
2	4	4	2	0	10
3	1	1	0	0	2
>3	3	2	2	0	7
Total	14	9	4	1	28

## Discussion

The present investigation showed that the overall seropositivity for *T. gondii *exposure was 10.81% in pet dogs in Lanzhou, which was lower than the values of 17.5% in dogs in a study performed in Guangzhou [15], 12.26% in Zhengzhou [18], and 13.21% in Beijing [25], but higher than those observed in Inner Mongolia [16], Haiko [23] and Shenzhen [17]. Among these regions, the difference in *T. gondii *seroprevalence may be due to ecological and geographical factors, as well as feeding and animal welfare conditions for dogs in these areas.

It is known that the average annual rainfall of Lanzhou is only 360 mm, and the average annual temperature is 9.3°C, with a typical continental monsoon climate. Dry and cold circumstances may be a challenge for the survival of *T. gondii *oocysts, and unfavorable for epidemics of toxoplasmosis.

In comparison with other age groups of dogs, a higher prevalence of infection was detected in the group of dogs >3 years old. Although the difference was not statistically significant among age groups (*p >*0.05), there is a general tendency for older animals to have had more exposure to *T. gondii*. Older animals have had more opportunities to come into contact with felids, so they may be more likely to acquire *T. gondii *infection by ingesting food contaminated with oocysts which have been shed and excreted in feces by cats. There were limited data on seroprevalence of *T. gondii *infection in cats in Lanzhou, our preliminary survey showed that the seroprevalence of *T. gondii *infection in pet cats in Lanzhou was 22.83% (unpublished data), indicating a high risk as a source of *T. gondii *infection in dogs and other animals and humans.

In addition to being infected by *T. gondii *oocysts shed and excreted in feces by infected cats, dogs may also become infected through ingestion of raw or uncooked meat/flesh contaminated with *T. gondii *cysts. Moreover, congential *T. gondii *infection in dogs is considered an important factor, the fetus may acquire initially *T. gondii *infection during pregnancy in female canines. A study found that reinfected pregnant female canines could transplacentally transmit *T. gondii *to their neonates [30]. Another study showed that dogs can vertically transmit *T. gondii *to their offspring by semen [31].

Statistical analysis showed that differences in *T. gondii *infection between female and male pet dogs were not significant (*p >*0.05), suggesting that gender of the host is not a crucial factor for *T. gondii *infection. In this study, we found a low (8.06%) prevalence of *T. gondii *infection in pet dogs in Anning District, compared with a seropositive rate of 9.52% in Qilihe District, 10.11% in Chengguan District and 13.79% in Xigu District, but statistical analyses showed that these differences were not significant (*p >*0.05).

In this study, we investigated the seroprevalence of *T. gondii *infection in 259 pet dogs from four districts in Lanzhou, northwest China between November to December, 2010. The number of pet dogs sampled was large enough to be representative, with the potential limitation that the results of the present investigation may not reflect the actual seroprevalence in other seasons, and in other districts of Lanzhou. Therefore, further comprehensive surveys of *T. gondii *infection in pet dogs in Lanzhou are warranted to sample more pet dogs during each climatic season and from all of the 8 districts and counties of Lanzhou. Also, *T. gondii *infection in stray dogs in Lanzhou will be considered in further investigations.

## Conclusions

The results of the present survey revealed a high prevalence of *T. gondii *infection in pet dogs in Lanzhou, especially in older pet dogs, which has public health significance because a previous survey of *T. gondii *infection in people with high risk of exposure to *T. gondii *in Lanzhou showed that 34.78% of pet dog owners were seropositive [32]. Therefore, it is necessary to take integrated strategies, including efficient management measures to prevent and control *T. gondii *infection in pet dogs, which could help to reduce *T. gondii *infection in other animals and humans in this area.

## Competing interests

The authors declare that they have no competing interests.

## Authors' contributions

XQZ and DHY conceived and designed the study, and critically revised the manuscript. BQF GYL, JXC, MXC and YBW participated in study design, study implementation and manuscript revision. SMW, SYH and DHZ performed the experiments, analysed the data and drafted the manuscript. BQF, GYL, JXC, MXC and ZGY helped in study implementation and data collection. All authors read and approved the final manuscript.
